# Experimental research on the relationship between the stiffness and the expressions of fibronectin proteins and adaptor proteins of rat trabecular meshwork cells

**DOI:** 10.1186/s12886-017-0662-5

**Published:** 2017-12-29

**Authors:** Chuan Wang, Lin Li, Zhicheng Liu

**Affiliations:** 1 Department of Biomechanics and Rehabilitation Engineering, School of Biomedical Engineering, Capital Medical University, No.10 Xitoutiao, You An Men, Beijing, 100069 People’s Republic of China; 2 Beijing Key Laboratory of Fundamental Research on Biomechanics in Clinical Application, Capital Medical University, Beijing, 100069 China; 3 YanJing Medical College, Capital Medical University, Beijing, 100069 China; 4 Department of Biomedical Informatics, School of Biomedical Engineering, Capital Medical University, No.10 Xitoutiao, You An Men, Beijing, 100069 People’s Republic of China

**Keywords:** TM cells stiffness, Intraocular pressure, AFM, Outflow resistance, Protein expression

## Abstract

**Background:**

Trabecular meshwork (TM) plays an important role in maintaining normal intraocular pressure (IOP). Studies have shown that glaucomatous TM tissues are stiffer than those of normal tissue. The high expression of fibronectin protein (FN) and adaptor protein (LNK) may be related to high resistance to aqueous humor outflow as well as high IOP. Our concern is what factors lead to the variation of the stiffness of trabecular tissue/cells.

**Methods:**

Atomic force microscope (AFM) and Western blot (WB) analysis were applied to test TM cells of rats cultured with different concentrations of dexamethasone (DEX) and mifepristone (MIF). Rat TM cells were randomly divided into 7 groups, marked as D1, D2, D3 and M1, M2 M3 for different concentrations of DEX and MIF, respectively, and C for blank control.

**Results:**

The elastic modulus of the treated cells were 2.67 ± 0.914 KPa, 2.92 ± 0.986 KPa, 4.52 ± 1.22 KPa for D1, D2, D3, 2.06 ± 0.745 KPa, 1.23 ± 0.462 KPa, 0.467 ± 0.275 KPa for M1, M2, M3, and 2.43 ± 0.713 KPa for C group, respectively. Expressions of FN and LNK increase (decrease) with the increase of the concentrations of DEX (MIF).

**Discussion:**

We focus on the relationship between the stiffness and the expressions of FN and LNK of rat TM cells. We analyzed the correlation between cell stiffness and FN, LNK expression, discussed the relationship between cell stiffness and aqueous humor outflow resistance.

**Conclusions:**

The changes of TM cell stiffness and the expressions of FN and LNK are positively correlated.

## Background

One of the important risk factors for glaucoma is high intraocular pressure (IOP) [1, 2] that is mainly caused by increased resistance to aqueous humor outflow within the conventional outflow pathway [3–5]. TM cells are the primary cell type that occupy and form the proximal portion of the conventional outflow pathway. There have been numerous studies on TM cells [6].

We have noticed that, on one hand, the increased production of extracellular matrix (ECM) components in the chamber angle by TM cells may be in part responsible for the development of increased IOP [7–9], the increased FN synthesis could result in a concomitant increase in IOP [10], and there is an abundance of collagen in TM samples of trabeculectomy specimens from glaucomatous eyes compared to those in the control samples [11]. These findings suggest that increased ECM deposition in the outflow pathway may cause resistance to aqueous outflow and high IOP. On the other hand, there are several observations which suggest that TM stiffness may play an important role in ocular hypertension associated with glaucoma. It is well known that TM cells are contractile [12]. The contraction of TM cell can direct ECM reorganization, and thus it has been hypothesized that the increased contraction state of TM cells in primary open-angle glaucoma (POAG) might be linked to a stiffer TM [13]. For example, pharmacologic modulation of TM cell actomyosin tone has a significant impact on aqueous outflow directly through the trabecular meshwork [14]. Further, it has reported that the compressive stiffness of TM was 20 times greater in post mortem glaucomatous human eyes compared to ostensibly healthy eyes [15].

Here we explicitly note that from one point of view, clinical drug treatment can change the IOP. In steroid-induced glaucoma, ECM component synthesis is increased and is associated with elevated IOP. Studies have reported that glucocorticoids, such as dexamethasone (DEX) can induce excess accumulation of ECM proteins in TM tissues and result in resistance to aqueous outflow and contribute to increased IOP [16–18]. Animal experiments confirmed that the glucocorticoid antagonist mifepristone (MIF) inhibits FN protein deposits. Thus reducing the normal rabbit IOP [19, 20]. From another point of view, drugs treatment can change the TM cell structure as well as the protein expression. Research has confirmed that LM and Col synthesis increase in cells subjected to high glucose (30 mM) or 0.1 mM DEX in bovine and human TM cells. Increased level of LM and Col protein resulted in reduced cell monolayer permeability [21, 22]. Studies have demonstrated that some of the glucocorticoid such as dexamethasone (DEX) is associated with specific morphological changes in the TM [23–25]. Depending on the existing research, drugs can regulate IOP, at the same time, the TM cell’s structure; protein expression has the corresponding influence [26–30].

Taking all of the above into consideration. We believe that thorough understanding TM cells, on the basis of protein expression and mechanical properties, can further allow us unveil the formation of aqueous humor outflow resistance. Our hypothesis is that FN and LNK protein expression may correlate with rat TM cell stiffness. If this hypothesis is verified, on the one hand, rat TM tissue/cell stiffness can be alternated by adjusting the level of protein expression, on the other hand, it allows to further explore the relationship between stiffness changes of trabecular tissue/cells and aqueous outflow resistance. The trabecular meshwork consists of endothelial cells immersed in their fundamental substance. The most notable characteristic is that these cells can change their shape, and changing their shape changes their gene expression [31].

If we use drugs to regulate TM cells protein expression, at the same time, test cell stiffness after drug treatment, then we can find whether there is a certain connection between them. The purpose of this study was intended on the relationship between FN and LNK protein expression and TM cell stiffness. In our experiments, AFM and WB were applied to test TM cells of rats cultured with different concentrations of DEX and MIF. For each group of TM cells cultured by a concentration gradient of DEX and MIF, FN and LNK expression data and TM stiffness were recorded. A correlation between TM cell stiffness and FN and LNK expression will be understood. This study has a progressive significance for explaining the formation of aqueous humor outflow resistance and the elevated IOP from the aspect of bio-mechanics of TM cells.

## Methods

### Material preparations

**Fig. 1:**
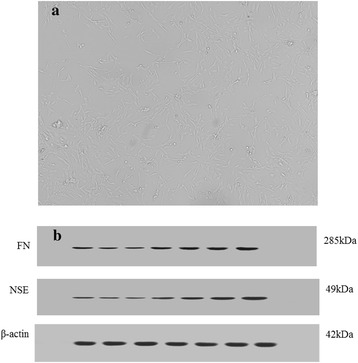
The rat trabecular meshwork cells (**a**) and cell type identification (**b**), spindle cells cultures confirm to rat TM’s morphological and WB results conform to the characteristic of the cell type

Authors state that they adhered to the tenets of the Declaration of Helsinki or the NIH statement for the use of Animals in Research. This research was approved according to relevant laws and institutional regulations. The Rat TM cells were supported by YanYu trade co., LTD. Shanghai, China. The supplier’s catalog number for the cells is FS02436. The primary cultures were established by those cells migrating from the excised Rat trabecular tissue [32]. Spindle cells cultures generated confirmation to progress through morphological and biochemical changes characteristic of the cell type (see Fig. 1.). Further, rat TM cells were identified and assayed by WB analysis with rabbit anti rat FN and NSE antibody (see Fig. 1.). In our experiment, the third generation of TM cells was selected as the experimental object.

Cell samples were homogenously inoculated in Petri dishes. After 12 h for cell adherent, Petri dishes were randomly divide into seven groups, each group two dishes. We marked them as D1, D2 and D3 for DEX (dexamethasone; Solarbio; Beijing; China) concentration at 10^−6^ M, 10^−5^ M and 10^−4^ M and M1, M2 and M3 for MIF (mifepristone; abcam; Cambridge; UK) concentration at 10^−6^ M, 10^−5^ M and 10^−4^ M. The other two dishes were marked as C for blank control group. After 48 h of cell cultivation, seven cells were randomly selected from each Petri dish for AFM cell mechanics test. We obtained cells force curve. The other group cells were used only for WB analysis. The expression of FN and LNK protein was recorded.

### Western blot analysis

The total protein was extracted with the RIPA (Applygen, Beijing, China) from TM cells subjected to different experimental conditions. The concentration of total proteins was monitored with BCA Protein Assay Kit (Dinao; Beijing; China). The proteins were diluted with RIPA buffer to the desired concentration and then mixed with an appropriate amount of loading buffer (Thermo, Pittsburgh, USA) with the proportion of 5:1. The protein mixture was taken to boil for 5 min at the temperature of 9 °C. The protein mixture (20 μg each lane) were separated using 10% SDS-PAGE gel electrophoresis (Beyotime Biotechnology, China) at a constant voltage of 80 V for 2.5 h at 4 °C and transferred to PVDF membranes (Millipore, USA) at a constant current of 250 mA for 1 h at 4 °C. And incubated at 4 °C overnight with the following primary antibodies: Anti-Fibronectin antibody, Anti-LNK antibody, Anti-NSE antibody (Abcam, Cambridge, UK). Membranes were incubated with 1:2000 dilution of peroxidase-conjugated goat anti-mouse IgG secondary antibody (Applygen, Beijing, China) for 1 h at room temperature. Finally, the membranes were incubated in ECL reagent (Pierce, Thermo, USA) for HRP detection and then exposed to autoradiography film (Bio-Rad, USA) for band visualization.

In our tests β-actin was used as an internal control. In fact, studies had tested the effects of 0.1 mM DEX for 10 days and found no significant change in β-actin protein expression in TM cells [33, 34]. Since both DEX and MIF are counteractive pattern with glucocorticoid receptors and the action time is short. We speculate that β-actin protein expression will not change together. We did three separated WB analysis for statistical analysis.

### AFM experiments

By measuring indentation at a given force, the local stiffness of the sample in terms of Young’s modulus can be extracted from the recorded force curve, where a model for the tip–sample contact mechanics [35–38] and the Hertz model [39] were applied. The force curve consists of an approach–retract cycle between the tip and the sample during which the cantilever deflection is measured as a function of the relative motion [40]. Elasticity information on a biological sample can be obtained by exploiting the loading force curve [41].

**Fig. 2:**
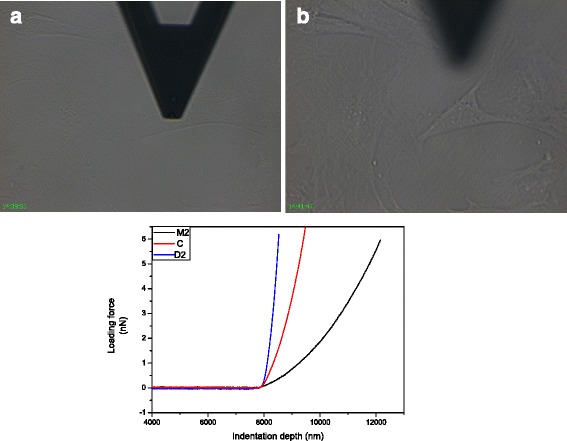
AFM Test for TM cells. One single TM cell was under indentation perpendicular to the nuclei surface (**a**) and after indentation (**b**). We obtained Force curve for each cell and showed three represented curves (below). Three curves represent cells in D2, M2, and C after translation the contact point at 8000 nm, the blue, dark and red line represents the D2, M2, and the control samples, respectively

Based on literature, where 1–3 cells [42], 6 cells [43] were applied to measure the stiffness by AFM, in this study, 7 single cells were randomly selected from each Petri dish for the atomic force microscope (AFM, NTEGRA, NT-MDT, Russia) cell mechanics test. We adopted spherical probe, probe models is MLCT - O10 - A, Bruker, sphere radius *r* = 2500 nm, cantilever spring constant k = 0.05 N/m, loading speed is 500 nm/s. When the spring constants of the cantilever and the sample are comparable, once the tip is in contact with the sample, upon further approaching the sample will undergo indentation, resulting in a minor cantilever deflection. Then the indentation force can be obtained. Indentation depth should select about 1/10 the thickness of the sample because a very thin sample may cause the tip to feel the underlying stiff substrate. In order to guarantee the consistency and certainty of the test, the axis along which the force is administered by the AFM tip is perpendicular to the nuclei surface to avoid lateral forces (see Fig. 2).

We can choose cells under the inverted microscope. For each cell the loading force curve and unloading force curve were obtained. The loading force curve for each cell was used to determine the elastic modules. The typical force curves were shown in Fig. 2, where the horizontal axis shows the indentation depth. The vertical axis shows the force to load. Since the contact points of the AFM tip and the cells were different, we did the translation process and all contact point at 8000 nm, and this process did not alter the stiffness value.

### The determination of cell stiffness

For cells, basically meet the assumption of Hertz theory, applies to the model of spherical tip [44]. Hertz model is most widely used for the characteristic of the elastic modulus of cells [45].

We used the Hertz model for loading force curve fitting and obtained young’s modulus E for each cell.

$$ F\left(\delta \right)=\frac{4\sqrt{R}}{3}\frac{E}{{\left(1-\nu \right)}^2}{\delta}^{\frac{3}{2}}+b,\kern0.75em \delta =x-a $$

**Fig. 3:**
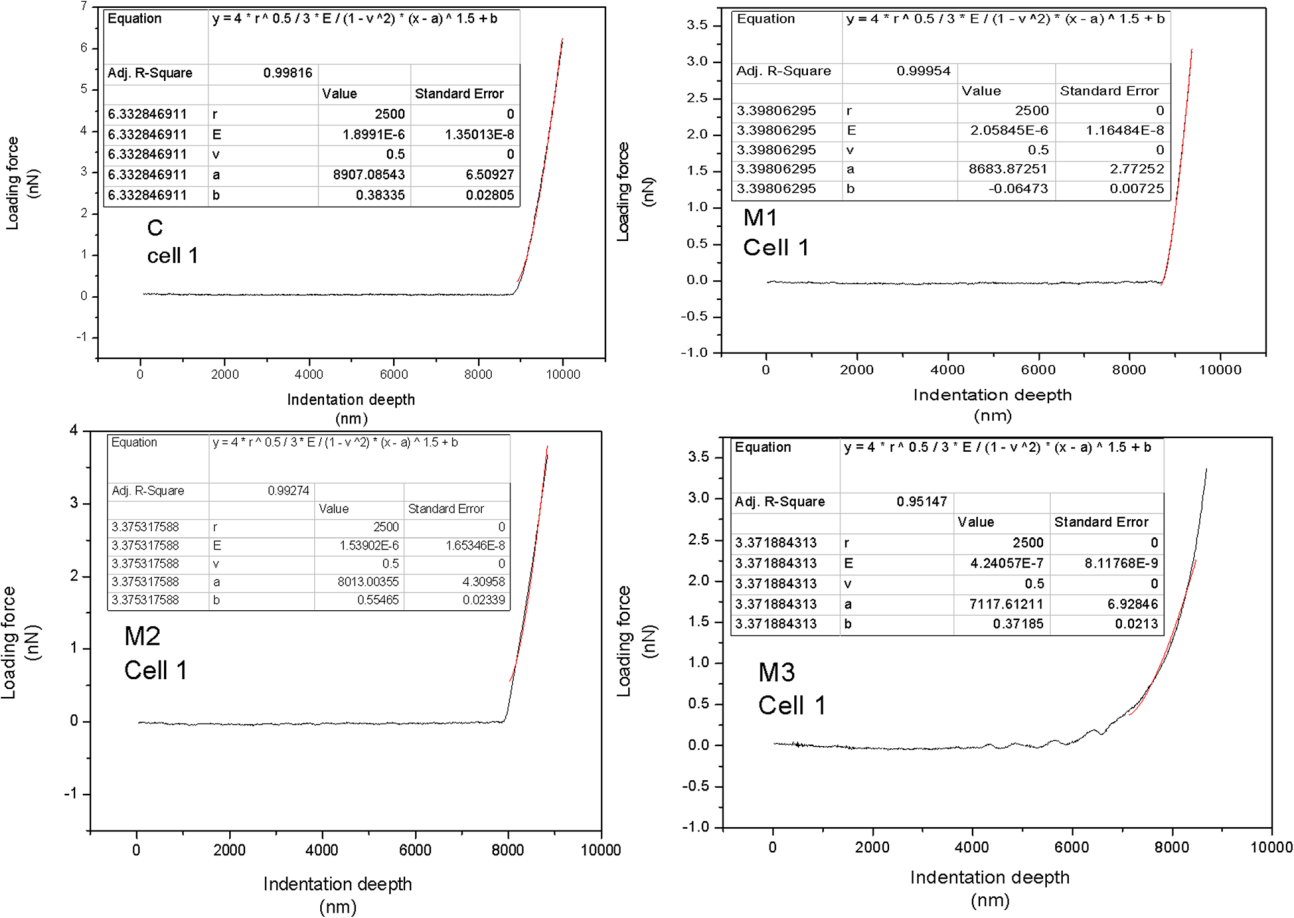
Curve fitting for each cell, the dark line is the loading force curve; we chose range about 1000 nm from the contact point for Hertz curve fitting, as shown in red line. The parameters are showed in the top left corner of each figure

The formula *F* represents the force (nN) applied to the sample, and *v* is poison ratio, typically take in biological samples *v* choose 0.5. *E* (GPa) represents elastic modules.*δ*(nm) represents indentation depth. In our curve fitting, sphere radius *R* = 2500 nm, poison ratio is 0.5, *a* and *b* represent the start point for curve fitting. We applied the Hertz equation for non-linear curve fitting to obtain the *E* value. This process and results were shown in Fig. 3.

## Results

**Fig. 4:**
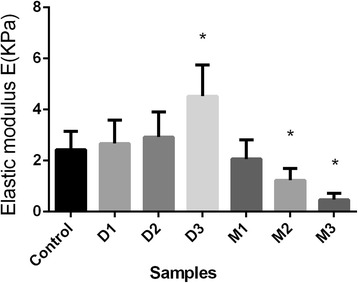
Results of standard one-way analysis of variance (ANOVA) compared to control group of cell stiffness. Significance was set at the probability (p) level of 0.05(5%), that is *p* = 0.05 (5%). The elastic modulus have significant changes (*) in D3, M2 and M3, by multiple comparisons

Table 1 gave the elastic modulus of the treated cells for each dish, expressed as means ± s.d. (standard deviations, *n* = 7). The elastic modulus of the DEX treated cells were 2.67 ± 0.914 KPa, 2.92 ± 0.986 KPa, 4.52 ± 1.22 KPa for D1, D2, D3, which was higher than the control group (2.43 ± 0.713 KPa). Oppositely, the elastic modulus of the MIF treated cells were 2.06 ± 0.745 KPa, 1.23 ± 0.462 KPa, 0.467 ± 0.275 KPa for M1, M2, M3, which was lower than the control group (2.43 ± 0.713 KPa). Furthermore, results of standard one-way analysis of variance (ANOVA) compared to control group of cell stiffness were shown in Fig. 4. Significance was set at the probability (p) level of 0.05(5%), that is *p* = 0.05 (5%). We did multiple comparisons between different groups. In D3, M2 and M3, elastic modulus have significant changes. Therefore, compared with the D3 group, TM cell stiffness can change about tenfold compared to the M3 group, and with the increase of DEX (MIF) concentration, TM stiffness increased (decreased).

**Table 1:** Cell stiffness for each sample

	Cell stiffness(E = Mean ± SD)		Goodness of fit (R^2^ = Mean ± SD)	
	Mean(KPa)	SD(KPa)	Mean	SD
C	2.43	0.713	0.998	0.001
M1	2.06	0.745	0.975	0.062
M2	1.23	0.462	0.986	0.018
M3	0.467	0.257	0.983	0.015
D1	2.67	0.914	0.998	0.002
D2	2.92	0.986	0.976	0.026
D3	4.52	1.22	0.973	0.031

**Fig. 5:**
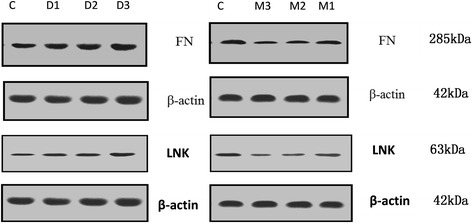
One of WB results of FN and LNK expression, FN and LNK expression up-regulated in D1, D2 and D3 group, in contrast, FN and LNK expression down-regulated in M1, M2 and M3 group. β-actin was used as an internal control

The results of one of WB analysis were shown in Fig. 5, where β-actin was used as an internal control in our studies. From Fig. 5, we got that the expressions of FN and LNK of the treated cell’s with D1, D2, D3 were up-regulated compared to control group. Reversely, M1, M2 and M3 treated cell’s FN and LNK expressions were down-regulated compared to the control group.

**Fig. 6:**
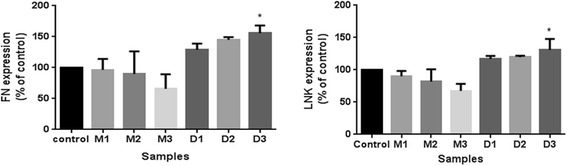
Result of FN expression (left) and LNK expression (right) compared to control group by one-way analysis of variance. Both M3 and D3 groups had significant alternation

The results of standard one-way analysis of variance (ANOVA) were shown in Fig. 6. WB gray analysis showed that with the increase of drug concentration DEX, FN/β-actin ratio is increasing, especially for D3 group, it increased by 55.58% compared with control group. In our AFM test D3 group had a significant cell stiffness increase compared with control group. With the increase of drug concentration of MIF, FN/β-actin ratio decreases. Similar changes were found in LNK protein expression. The significant increase was about 30.98% compared with control group. The changes in the two kinds of protein expression that alters the ECM and intracellular components and might further lead to the change of cell structure and mechanical properties such as the stiffness change.

## Discussion

This study applied atomic force microscope (AFM) and western blot (WB) technique to test TM cells of rats cultured with different drug concentrations of DEX and MIF and investigated the relationship between the changes of TM cell stiffness and the expression of fiber connection protein (FN) and adaptor protein (LNK).

Firstly, our results showed that the ranges of elastic modulus were 0.467 to 2.06 KPa for treated TM cells by MIF, 2.67 to 4.52 KPa for DEX treated TM cells, and 2.43 ± 0.713 KPa for the C group. It has reported that basal cell stiffness values were 3.71 ± 0.37 KPa, 4.33 ± 3.07 KPa (median ± S.D.) for cells derived from donors [46, 47]. It can be considered that the two results have good consistency. Secondly, DEX treatment resulted in a slight increase in the level of FN expression in D1 and D2 group, which was further enhanced in D3 group. This was consistent with published results [47]. And thirdly, down-regulate FN and LNK expression results were found in MIF treated group. This can decrease cell stiffness in turn. Mifepristone is a synthetic progesterone antagonist that influences Endoplasmic reticulum (ER) stress response. ER is the sites of protein synthesis and processing as well as cell signal processing. Endoplasmic reticulum stress directly affects the outcome of cellular stress, such as adaptation, damage or apoptosis [48]. To the knowledge of the authors, there have been no reports on FN and LNK expression after treatment of MIF available yet. All information above elucidate that the FN and LNK protein expression have a positive correlation with the stiffness of the TM cells.

### The correlation between cell stiffness and FN, LNK expression

FN protein is a hot spot of research for TM cells. FN protein plays a major role in cell morphology and function. It is discovered abundantly in TM beams, with slightly higher levels in the basement membranes of TM beam cells [49, 50]. Stiffness of TM cells after treated with DEX has different degree increase (2-fold for human patients TM [51], 3.5-fold for rabbit TM [52], and more than 2-fold for rat TM in this study). It develops a fibril with considerable elasticity [52, 53]. The increase of FN means elastic fibers increased in the ECM, and thus enhanced the elastic modulus of the cell. Besides, our elastic modulus result was derived from AFM probe direct perception of cells, cell stiffness is closely linked to the measured elastic modulus. DEX (MIF) treated cell’s FN expression increase (decrease), the corresponding elastic modulus also increase (decrease) accordingly. The stiffness alternation may correlate with the expression of FN protein.

LNK is an adaptor protein. It is an important regulator of TM cell kinetics, including the ability to cell growth, mobilization, and recruitment for cell regeneration [54]. Its function involves endothelial cell activation, regulation and control of the cytoskeleton. It also involves adjusting its structural characteristics. Signal transduction mechanism and biological function. It can be compatible with the formation of macromolecular compounds, to participate in the integration and intracellular signal transmission. It can be speculated that the increased amount of LNK expression makes the above functions enhanced; as a result, increase macromolecular compounds may have a close relationship with cell stiffness increase.

### The relationship between cell stiffness and aqueous humor outflow resistance

It has reported that aqueous humor outflow resistance may increase by DEX treatment [55]. Study reported that after topical administration of 0.1% DEX in vivo for 3 weeks, the elastic modulus of TM in D-treated eyes was 3.89 ± 2.55 kPa, which was significantly larger than that in control eyes. Therefore, by DEX treatment both TM cell stiffness and aqueous humor outflow resistance increase. Opposite to DEX [56], MIF is a kind of cortical antagonist, may occur the opposite effect. We found that MIF can dramatically decrease cell stiffness. If by MIF treatment aqueous humor outflow resistance decrease, we can assert that cell stiffness and aqueous humor outflow resistance are positively correlative. In fact, MIF treatment can lower IOP [57, 58]. On the other hand, our results indicate that the expressions of FN and LNK decrease after MIF treatment. It follows that ECM deposition in the TM reduces. This may decrease the aqueous humor resistance through the trabecular meshwork. During glaucoma, high IOP may be correlated with rat TM cell’s oxidative stress. Oxidation may lead to TM endothelial cell decay, tissue malfunction and subclinical inflammation, these changes reduced TM outflow facility and resulted increased IOP [48]. The mechanisms responsible for the aqueous humor that increases during glaucoma are different affecting the trabecular cells and inducing an IOP increase. According to the discussion above, we infer that there is a positive correlation relationship between the change in cell stiffness and aqueous outflow resistance. This assertion needs to be further studied by combining with the animal in vivo experiments.

#### Limitations

TM cells should be regarded as a kind of viscoelastic materials. The relationship between the viscoelastic properties of TM cells and the expressions of FN, LNK and/or other proteins, as well as aqueous outflow resistance has remained unclear. TM cells have the ability to secrete the ECM, which expresses proteins and cytokines [31]. There is no causal or mechanistic study done in order to determine the role of FN and LNK in TM cell mechanics.

Living cells will change its mechanical response according to mechanical stimulation [31]; however, this is inevitable for our experiment test. Our interpretation is that under the action of the two drugs, for the TM cells, cells may lose their ability to adapt to the mechanical stimulation. The metabolic and homeostatic conditions of TM cells are changing with DEX and MIF. The change of cell biology will lead to cell mechanics properties change. The presented data showed a correlation between FN and LNK protein expression and TM cell stiffness. It needs further to study the association of active and inactive forms of FN (LNK) with biomechanics.

There is growing evidence suggesting that mechanical properties of TM may be involved in ocular hypertension associated with glaucoma. We expect that the approach shown in this article will be used in future work to study issues such as identification of molecular factors and associated genes involved in TM stiffness regulation. Progress in this area may lead to further understanding of the role of TM stiffness in ocular hypertension, which may help guide the design of future glaucoma therapies.

## Conclusions

By using AFM we got TM cells modulus of elasticity treated by DEX and MIF, and analyzed the stiffness changes from the angle of molecular biology of FN, LNK protein expression. We found that cell stiffness changes and FN, LNK expression has a positive correlation, although the relationship between the expression of protein and the change of cell stiffness has not been clear yet. The results can further reveal the relationship between the organization and the change of mechanical properties of TM tissue and aqueous humor outflow resistance.

## Abbreviations

AFM: Atomic force microscope
DEX: Dexamethasone
E: Elastic modulus
ECM: Excetral cellular matrix
FN: Fibronectin protein
IOP: Intraocular pressure
LNK: Adaptor protein
MIF: Mifepristone
POAG: Primary open-angle glaucoma
TM: Trabecular meshwork
WB: Western blot
